# Effects of antibiotics (enrofloxacin) on microbial community of water and sediment in an aquatic ecological model

**DOI:** 10.3389/fvets.2023.1151988

**Published:** 2023-05-30

**Authors:** Yue Dai, Jin-Ju Peng, Teng-Yue Zhang, Xing-Peng Xie, Shuai-Shuai Luo, Wen-Chao Liu, Yi Ma

**Affiliations:** ^1^College of Coastal Agricultural Sciences, Guangdong Ocean University, Zhanjiang, China; ^2^Maoming Branch, Guangdong Laboratory for Lingnan Modern Agriculture, Maoming, China

**Keywords:** aquatic environment, aquatic ecological model, bacterial community structure, enrofloxacin, 16S rDNA

## Abstract

In order to explore the impact of antibiotics (enrofloxacin) on microbial community in aquatic environment, an indoor aquatic ecological model was built, and different concentrations of enrofloxacin (0.05, 0.5, 5, and 50 mg/L) were added in the aquatic ecological model. In addition, the water and sediment samples were collected on the 0, 7, 30, and 60 days, and the changes in microbial community were studied through 16S rDNA high-throughput sequencing. The results showed that when the concentration of enrofloxacin was 50 mg/L, the relative abundance of *Actinomycetes* was increased. In the water, the bacterial richness and diversity communities first decreased and then gradually recovered with the passage of time; On the 7th day, the diversity and richness index of species in the treatment groups with enrofloxacin at 5 and 50 mg/L decreased to the lowest; On the 30th day, the diversity and richness index of species began to rise; On the 60th day, the diversity index and richness index of water species began to increase, while the diversity index and richness index of sediment species decreased. In conclusion, the addition of enrofloxacin negatively affected the microbial community structure in an indoor aquatic ecological model, 50 mg/L enrofloxacin could increase the relative abundance of *Actinomycetes,* and decrease the diversity and richness index of water and sediment.

## Introduction

Antibiotics are widely used in the fields of medicine, aquaculture, and animal husbandry as pathogenic microorganism inhibitors and/or growth promoters during recent decades (1). However, about 30–90% of antibiotics in humans and animals are discharged into the body through urine and feces, which are often not fully utilized and treated, resulting in a large number of residues of antibiotics and their metabolites in the soil, water, and vegetation of the environment (2–4). It has been shown that the concentration of antibiotic residues directly affects the structure and community of microorganisms in the ecosystem (5). Enrofloxacin is a kind of fluoroquinolone antibiotics, which has strong antibacterial activity against most gram-negative bacteria, some gram-positive bacteria and mycoplasma (6, 7). After the non-covalent combination of two enrofloxacin molecules with DNA-topoisomerase complex (II or IV) near the tyrosine residue of the active site of bacteria, its induced enzyme underwent conformational changes, which led to the formation of enrofloxacin-rotatase/topoisomerase IV-DNA complex, and directly inhibited bacterial DNA replication (8, 9). Low concentration of enrofloxacin would inhibit bacterial DNA rotatase and topoisomerase IV, but high concentration of enrofloxacin can directly destroy bacterial chromosomes, leading to cell death (10, 11).

Because of the excellent and broad-spectrum antibacterial effect, enrofloxacin is frequently used as a therapeutic antibiotic and growth promoter in aquaculture, resulting in its metabolites remaining in the soil and aquaculture environment. According to the report of Li et al. (12), they detected nine kinds of quinolones in the seawater of aquaculture areas, and the concentration range of enrofloxacin residue was 6.91–17.49 ng/L. The main place where norfloxacin accumulates in aquatic environments is surface water, such as rivers, where the level is between 12 ng/L and 4.24 μg/L (13), and the concentrations of streams and ponds are about 17–216 ng/L (14) and 0.50 μg/L (15) respectively. Besides, it is reported that enrofloxacin with concentration ranging from 2.0 to 4.0 ng/L was detected in the tap water of Guangzhou and Macao (16). The largest residues of enrofloxacin are domestic wastewater, municipal wastewater and hospital wastewater, and the concentration of enrofloxacin may be as high as 100 μg/L. (17) Therefore, the toxicity of enrofloxacin to the ecological environment and its impact on the microbial community and structure in the environment are of great concern. The abuse of enrofloxacin in aquaculture has a negative impact on the aquatic ecological environment and may affect human public health through the ecological cycle (18). Microorganisms are an important part of the aquatic environment, which play an important role in the response to antibiotics, the spread of drug resistance, and environmental remediation (19). It has been found that the antibiotics could directly affect the drug resistance of environmental microflora, thus causing environmental toxicity (20, 21). However, the relevant reports about the impact of enrofloxacin on the microbial community in the aquatic environment are limited. Therefore, this study evaluated the effects of different concentrations of enrofloxacin on bacterial community in an indoor aquatic ecological model.

## Materials and methods

### Reagents

The lake surface water (1–10 cm) and lake surface sediments (1–10 cm) in Guangdong Ocean University (Zhanjiang, China) were collected, and the debris was removed to construct an aquatic microcosm. Enrofloxacin (99.95%) was purchased from Zhejiang Guobang Pharmaceutical Co., Ltd. (190720-2, Hangzhou, China). Water DNA Kit (D5525), Soil DNA Kit (D5625), and MicroElute Gel Extraction Kit (D6294) were obtained from Omega Bio-Tek Company (Guangzhou, China).

### Experimental design and sampling

The indoor aquatic ecological model was constructed according to our previous study, and the details of the aquatic ecological model have been reported previously (20). Briefly, the water and sediment used for constructing aquatic models are all taken from the same location of the artificial lake on the campus of Guangdong Ocean University (Zhanjiang, China). The 60 (length) × 50 (width) × 30 (height) cm transparent plastic boxes were used for constructing aquatic model. First, lay the sediment at the bottom of the plastic box, so that the height of the sediment is 10 cm, and then add 45 L of water. The illustration of the indoor aquatic ecological model is presented in Supplementary Figure S1. The experiment was divided into five groups, with three replicates in each group, a total of 15 aquatic model boxes were used. Enrofloxacin was added to the water at 0, 0.05, 0.5, 5, and 50 mg/L (water and sediment were W0–W4 and S0–S4, respectively), and was cultured at room temperature of 25 ± 3°C for 60 days. Samples of water (Group W) and sediment (Group S) were collected at 0, 7, 30, and 60 days (D0, D7, D30, and D60). The 5% sodium hydroxide solution was used as the solvent for enrofloxacin. The specific method for adding different concentrations of enrofloxacin is as follows: take 5 g of enrofloxacin, add an appropriate amount of 5% sodium hydroxide solution until it is completely dissolved, then add double distilled water to a constant volume until the concentration of enrofloxacin is 5 g/L. Then dilute it with double distilled water to several concentrations of 5, 50, and 500 mg/L, and then 30 L of water was taken from each aquatic ecological model. After discarding 450 mL of water, 450 mL of corresponding concentration of enrofloxacin solution was added. For the control group, 450 mL of double distilled water without enrofloxacin was added. So that the enrofloxacin concentrations in the water bodies of each model were 0, 0.05, 0.5, 5, and 50 mg/L.

### 16S rDNA sequencing and statistical analysis

The supernatant of water and sediment samples was removed by centrifugation, and the total genomic DNA of water and sediment microorganisms was extracted according to the instructions of the kit. The concentration and purity of extracted DNA were determined by Nanodrop UV–Vis spectrophotometer. The extracted DNA was sent to Suzhou Golden Wisdom Biotechnology Co., Ltd. for Illumina MiSeq sequencing. QIIME was used to optimize the original sequencing data: splicing the overlapping regions at the end of the sequence; Remove the sequence with length less than 200 bp; Remove the chimera sequence and get valid data. OTU cluster analysis was performed according to 97% similarity. The Silva 138 16S rDNA database was used for species classification annotation. The statistical analysis of the 16S rDNA sequencing data was reported in our earlier study (20).

## Results

### Microbial diversity analysis

The operational taxonomic units (OTUs) of bacteria were presented in Figure S2, the rarefaction curve of the OTUs was showed in Figure S3, and the unweighted unifrac distance matrix heat map was showed in Figure S4, and the principal coordinate (PCoA) analysis and principal component (PCA) analysis were presented in Figure S5 and S6. In addition, The community richness index mainly includes Chao1 index and ACE index, and the flora diversity index mainly includes Shannon index and Simpson index. As shown in Supplementary Table S1, among the sediment samples, the diversity index values of S4D60 sample are the lowest; As shown in Supplementary Table S2, in the water, the diversity index values of W3D7 samples are the lowest; It shows that enrofloxacin can inhibit the richness and diversity of bacterial species in S4D60 and W3D7 samples. With the increase of drug concentration in the water body, both the richness and diversity of the colony showed a downward trend. At a higher drug concentration, with the prolongation of the action time, the richness and diversity of the colony showed a downward trend and then gradually rose. At 30 days, the diversity index began to rise, and the higher the drug concentration, the smaller the index recovery trend.

### Analysis of bacterial abundance and distribution

**Figure 1:**
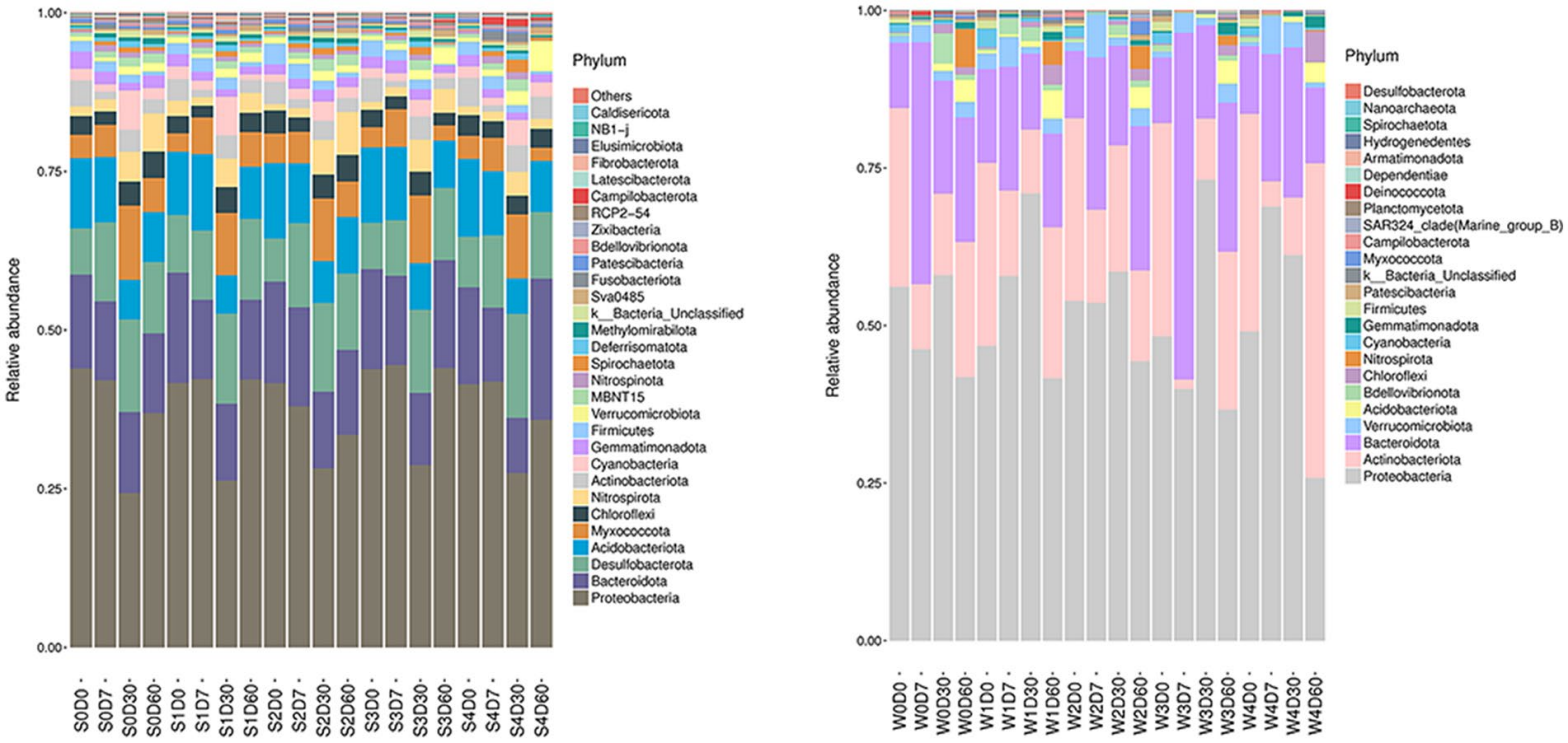
Effects of different concentrations of enrofloxacin on microbial abundance at the phylum level in an aquatic ecological model. W, water; S, sediments.

The analysis of non-metric multidimensional scale method (NMDS) and unweighted pair group method with arithmetical mean (UPGMA) was presented in Figure S7 and S8. The relative abundance of microbials was analyzed at the phylum level, and the information of the first 30 species is selected to draw a histogram as shown in Figure 1. The dominant bacteria in the water are *Proteobacteria, Actinobacteriota*, and *Bacteroidota*, and the dominant bacteria in the sediment are P*roteobacteria, Bacteroidota, Desulfobacterota, Acidobacter*, and *Myxococcota*. The water and sediment are the largest species of *Proteobacteria*, and the relative abundance of *Proteobacteria* in the water is 25–73%, and the relative abundance of *Proteobacteria* in the sediment is 24–44%. With the change of time, the relative abundance of *Proteobacteria* increased first and then decreased; the relative abundance of *Actinomycetes* and *Bacteroides* decreased first and then increased with time. In water, the relative abundance of *Bacteroides* in W3D7 samples increased; the relative abundance of *Actinomycetes* in W4D60 samples increased, while the relative abundance of *Proteobacteria* decreased. In the sediment, the relative abundance of *Myxococcus* in S4D60 samples decreased.

**Figure 2:**
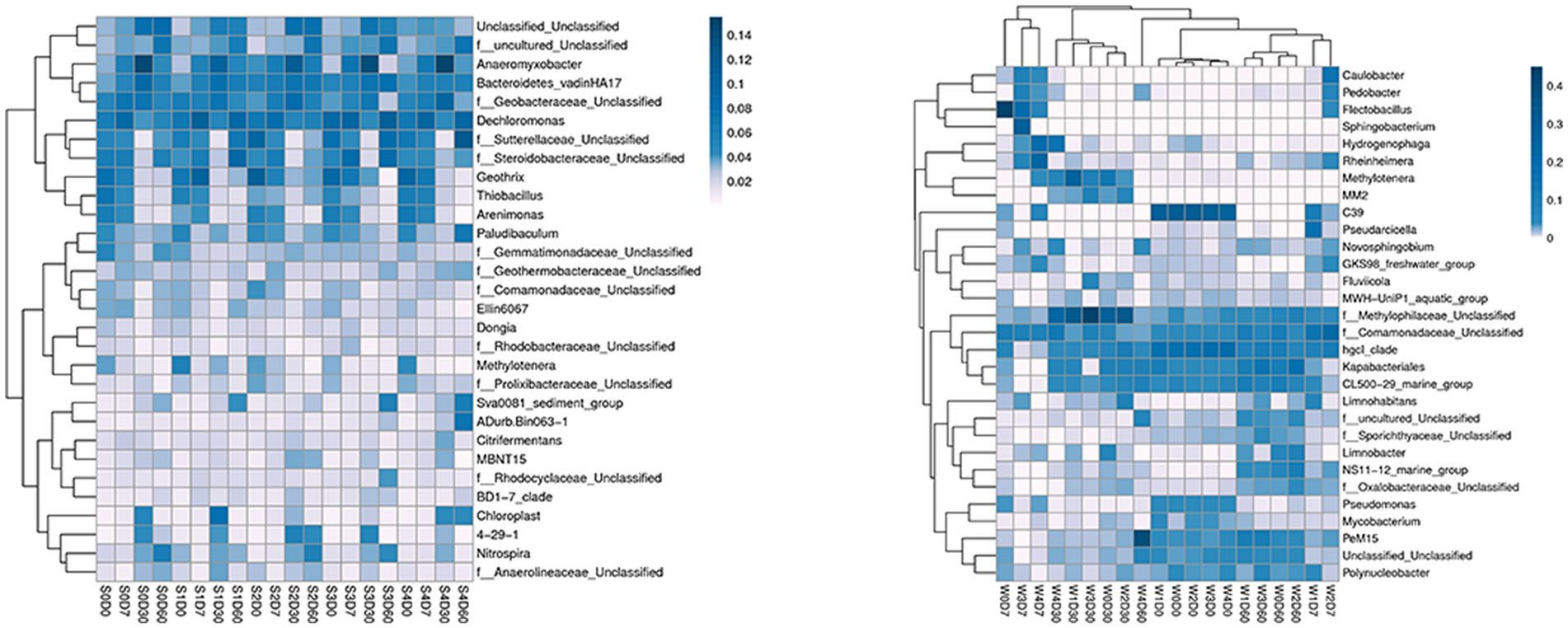
Effects of different concentrations of enrofloxacin on microbial abundance at the genus level in an aquatic ecological model. W, water; S, sediments.

At the genus level, a total of 413 genera were obtained from sediments, with the number of each group being 275–349, and 248 genera were obtained from water samples, with the number of each group being 117–181. The first 30 genera of horizontal species were selected to draw the Heatmap map, as shown in Figure 2. The main dominant genera in the water without enrofloxacin were *Proteobacteria C39*, *Chlorophyllaceae* and *Comamandaceae*; *HgCI-class, CL500-29-marine-group*, *Myxococcota, PeM15* of *Actinobacteriota*; *Pseudoricicella* of *Bacteroidota*, etc. In water, the relative abundance of *Actinomycete PeM15* in W4D60 samples increased. The main dominant bacteria in the sediment are *Dechromonas, Anaeromyxoactor, Bacteroidetes-vadinHA17, Geobactaceae, Geothrix, Steroidobactaceae*, etc.

## Discussion

There are a variety of microorganisms in the aquatic ecosystem, which play a key role in the aquatic environment, and carry out the circulation between material exchanges and maintain the stability of the water environment (22, 23). In recent years, the abuse of antibiotics has left a large number of residues in the aquatic environment, and the microorganisms in the aquatic environment will obtain the exogenous resistance genes through gene mutation, generation inheritance, transduction, conjugation, and transformation (24, 25). Some studies have also described that under the low concentration of antibiotics, microorganisms change the microbial community as a signal molecule so as to adapt to the residues of different concentrations of antibiotics in the aquatic environment (26–28).

In this experiment, the aquatic environment with enrofloxacin concentration less than 0.5 mg/L has no effect on the diversity and abundance of bacterial, while the concentration of enrofloxacin at 5 mg/L has a greater impact on bacterial community. Wei et al. (29) found that the increased stress concentration of enrofloxacin added to soil would significantly affect the soil bacterial community and change the ability of microorganisms to use carbon sources. In addition, fluoroquinolone antibiotics show resistance to hydrolysis and thermal coupling, and have good chemical stability, so there is a high degree of persistent pollution in the complex aquatic environment (30). In aquaculture, the antibiotics in the water will be degraded quickly due to the effect of light (31). However, when enrofloxacin and its metabolite ciprofloxacin enter the water body, 65% will be absorbed by the sediment (32). It has been suggested that the degradation of enrofloxacin is mainly photolysis, so enrofloxacin entering the sediment can be maintained for a long time, and can be slowly released into the water body through the desorption of the sediment (33). This partly explained that enrofloxacin had a greater impact on the diversity and abundance of microbial communities in water and a smaller impact on microbial communities in sediment in this experiment. In this study, we found that when the concentration of enrofloxacin was 50 mg/L, the relative abundance of *Actinomycetes* increased. According to previous studies, the presence of antibiotics in the aquatic environment will change the microbial community structure and diversity of the biofilm, and with the increase of antibiotic pollution concentration, the content of *Actinomycetes* in the aquatic environment will also increase (34). This indicates that high concentration of enrofloxacin can increase the relative abundance of drug-resistant bacteria. Therefore, in the aquaculture ecosystem, the residues of antibiotics will induce the microbial community to produce resistance genes, and the number of drug-resistant bacteria will increase significantly, polluting the drinking water source and animal-derived food, and posing a potential threat to human health (35).

In summary, with the increase of enrofloxacin concentration in the water, the richness and diversity of microbial community showed a downward trend. At a higher enrofloxacin concentration, with the prolongation of action time, the richness and diversity of bacterial community showed a downward trend and then gradually rose. The diversity and richness of microbial community in sediment are higher than that in water. The higher concentration of enrofloxacin added, the greater impact on the community and structure of microorganisms in the aquatic environment, and will increase the number of drug-resistant bacteria.

## Data availability statement

The datasets presented in this study can be found in online repositories. The names of the repository/repositories and accession number(s) can be found at: https://www.ncbi.nlm.nih.gov/, bioproject/926010.

## Author contributions

YM and W-CL: funding acquisition and writing—review and editing. YD: investigation. T-YZ and J-JP: methodology. X-PX and YD: project administration. S-SL: software. YM: supervision. YD and J-JP: writing—original draft. All authors contributed to the article and approved the submitted version.

## Funding

This research was supported by Guangdong Natural Science Foundation Project (2023A1515012181); Guangdong Provincial Department of Education 2021 Special Project for Key Fields of Ordinary Colleges and Universities (2021ZDZX4003); Zhanjiang Science and Technology Bureau 2021 Provincial Science and Technology Special Funds (“Big Special + Task List”) Competitive Allocation Project (2021A05231); and Start-up Research Project of Maoming Laboratory, Guangdong Laboratory for Lingnan Modern Agriculture (2021TDQD002).

## Conflict of interest

The authors declare that the research was conducted in the absence of any commercial or financial relationships that could be construed as a potential conflict of interest.

## Publisher’s note

All claims expressed in this article are solely those of the authors and do not necessarily represent those of their affiliated organizations, or those of the publisher, the editors and the reviewers. Any product that may be evaluated in this article, or claim that may be made by its manufacturer, is not guaranteed or endorsed by the publisher.
