# Supplementary figures and images for: Effects of antibiotics (enrofloxacin) on microbial community of water and sediment in an aquatic ecological model
Source: Front Vet Sci. 2023 May 30;10:1151988. doi: 10.3389/fvets.2023.1151988 (PMC10267828; doi:10.3389/fvets.2023.1151988)

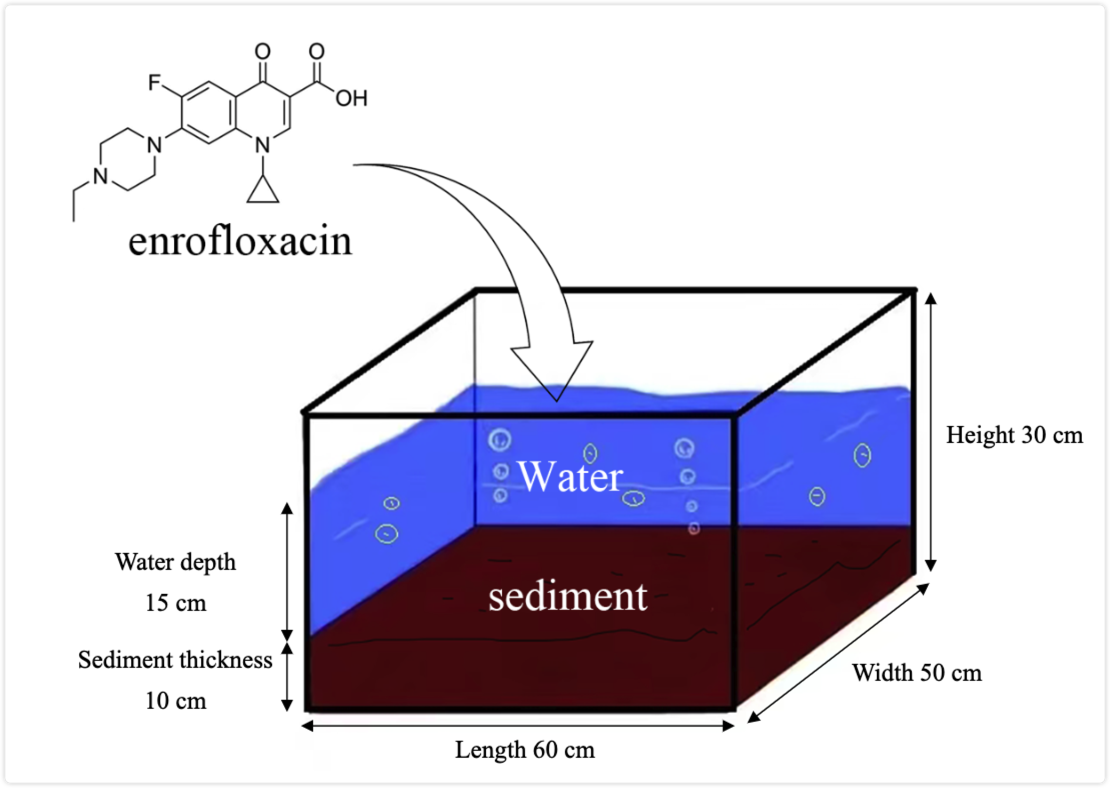

Supplement: Supplementary file 1 [file Image_1.TIF]

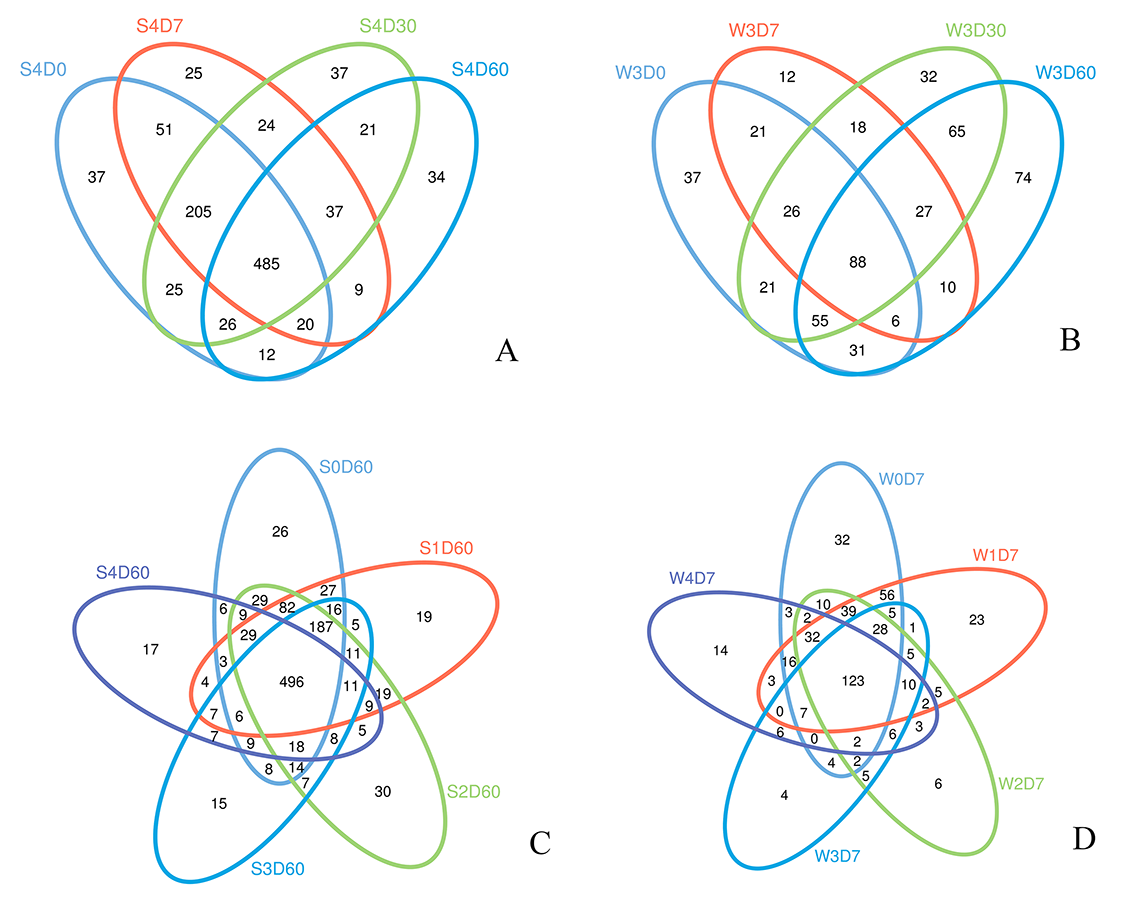

Supplement: Supplementary file 2 [file Image_2.TIF]

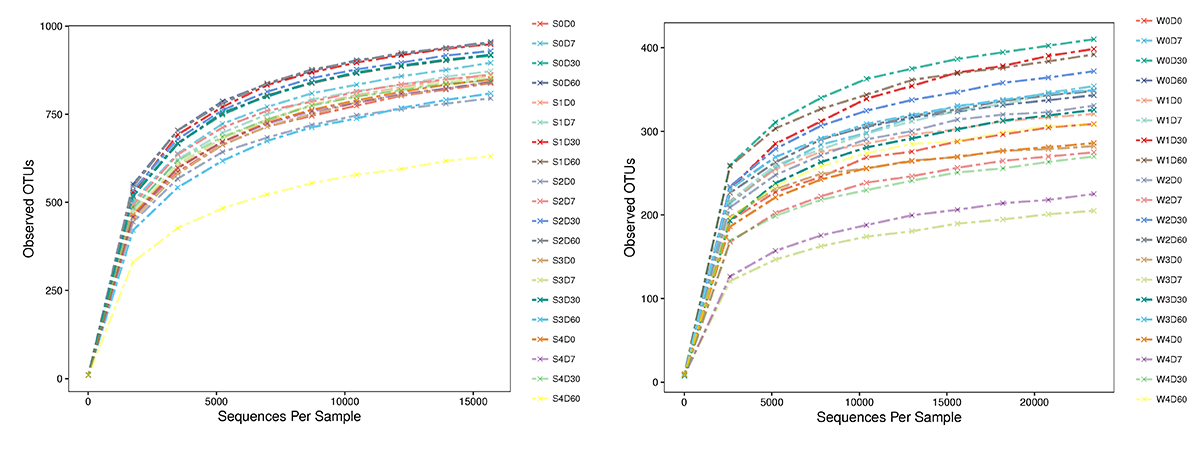

Supplement: Supplementary file 3 [file Image_3.TIF]

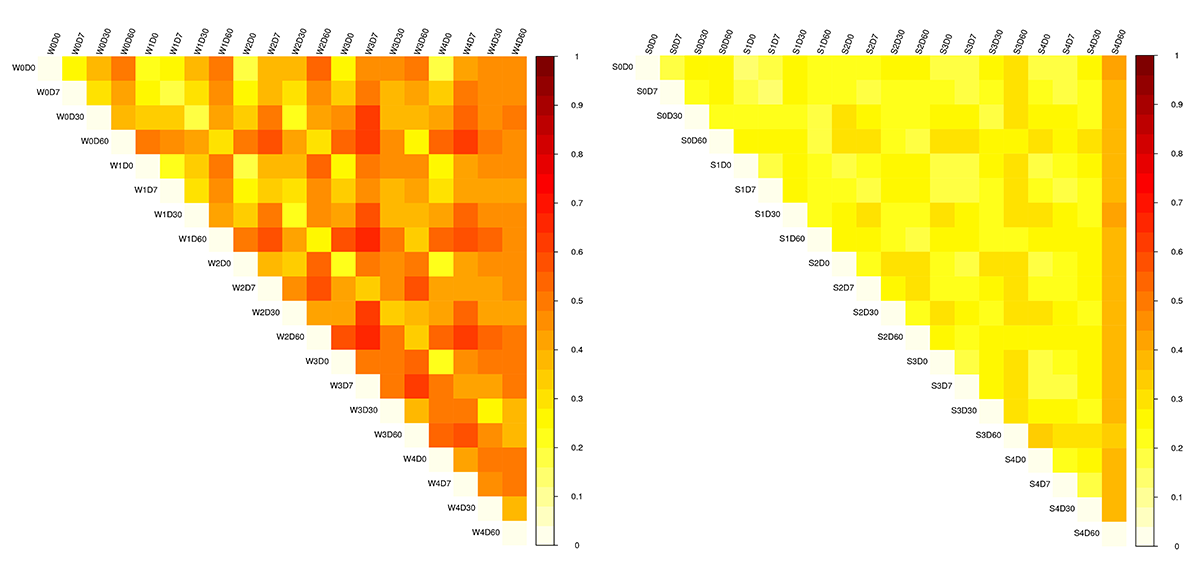

Supplement: Supplementary file 4 [file Image_4.TIF]

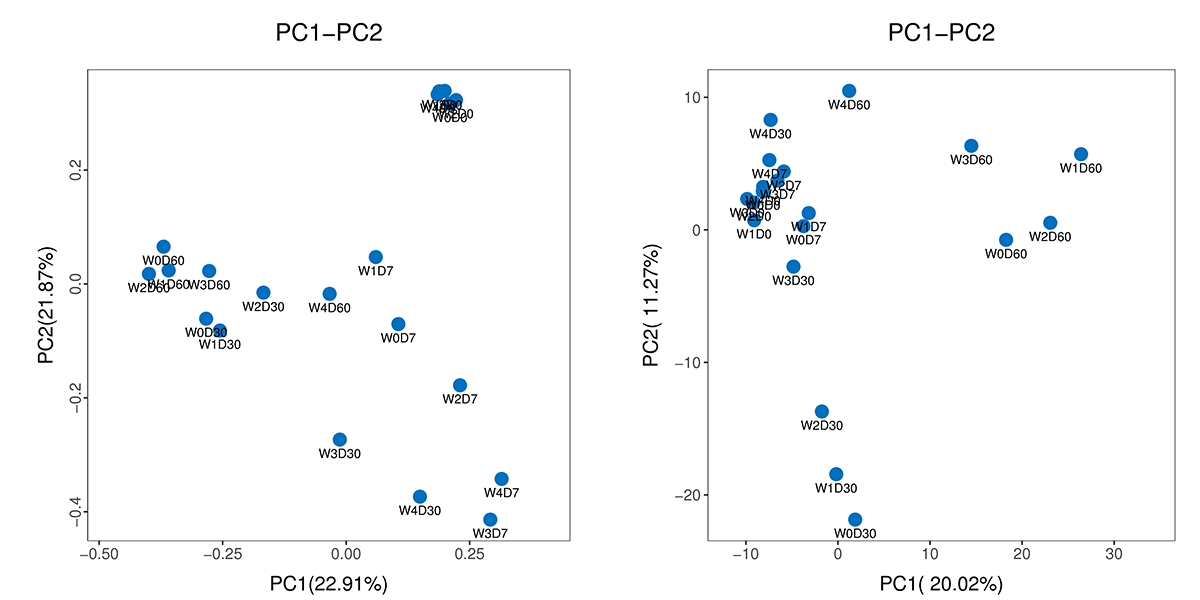

Supplement: Supplementary file 5 [file Image_5.TIF]

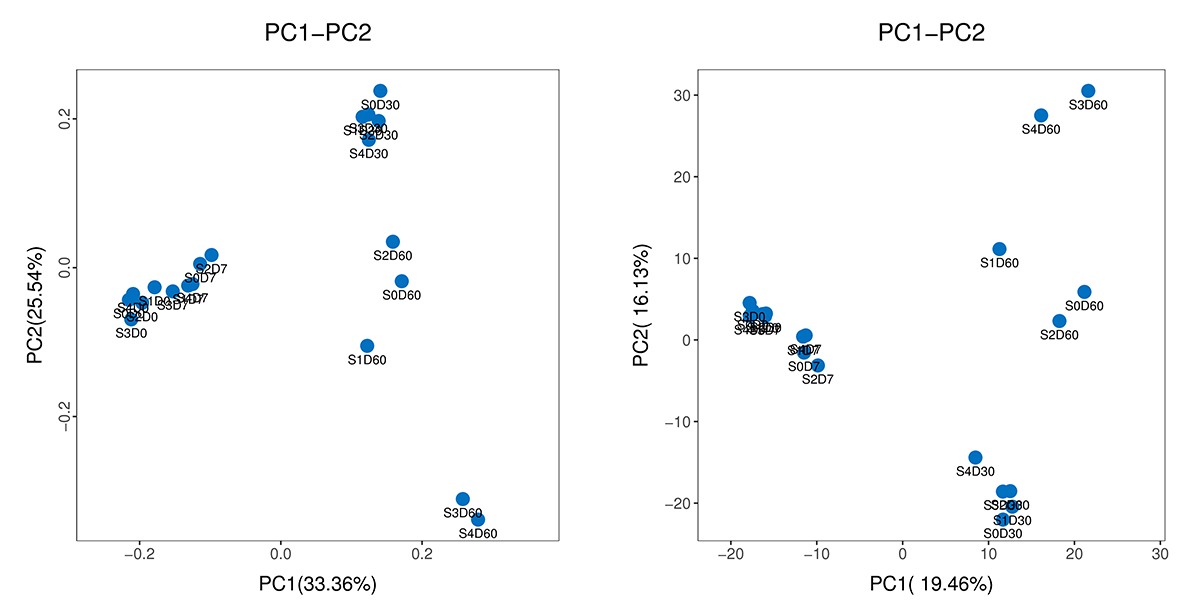

Supplement: Supplementary file 6 [file Image_6.TIF]

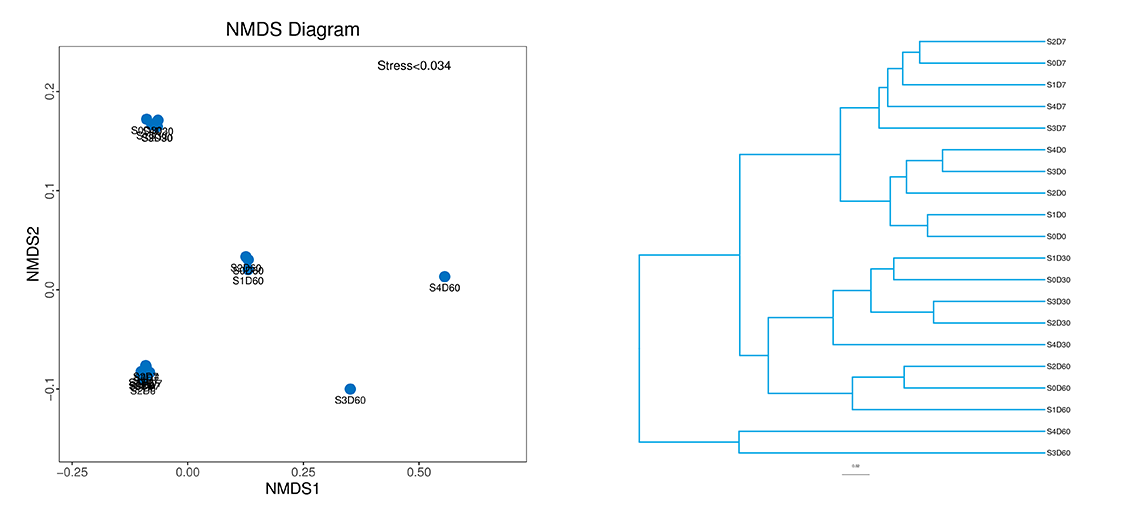

Supplement: Supplementary file 7 [file Image_7.TIF]

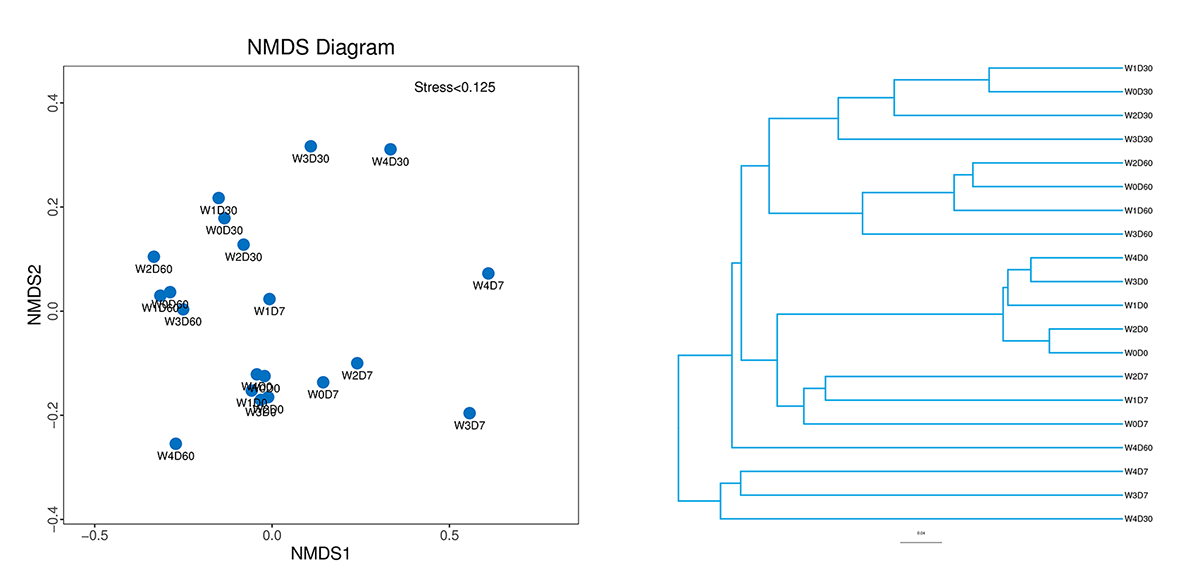

Supplement: Supplementary file 8 [file Image_8.TIF]
