# Supplementary material for: Effects of antibiotics (enrofloxacin) on microbial community of water and sediment in an aquatic ecological model
Source: Front Vet Sci. 2023 May 30;10:1151988. doi: 10.3389/fvets.2023.1151988 (PMC10267828; doi:10.3389/fvets.2023.1151988)
Supplement: Supplementary file 9 [file Table_1.DOCX]

**Table S1.** Alpha diversity index of bacteria in sediments

| Sample | ACE | Chao1 | Shannon | Simpson |
| --- | --- | --- | --- | --- |
|  |  |  |  |  |
| S0D0 | 959.884 | 978.614 | 8.343 | 0.994 |
| S0D7 | 980.894 | 993.598 | 8.536 | 0.995 |
| S0D30 | 974.414 | 981 | 8.656 | 0.995 |
| S0D60 | 1020.625 | 1026.641 | 8.84 | 0.996 |
| S1D0 | 962.88 | 970.727 | 8.447 | 0.995 |
| S1D7 | 959.779 | 976.57 | 8.387 | 0.994 |
| S1D30 | 1014.688 | 1024.978 | 8.701 | 0.996 |
| S1D60 | 988.893 | 993.938 | 8.53 | 0.994 |
| S2D0 | 865.314 | 877.785 | 8.188 | 0.992 |
| S2D7 | 928.199 | 942.909 | 8.567 | 0.995 |
| S2D30 | 986.796 | 986.03 | 8.752 | 0.996 |
| S2D60 | 1016.488 | 1035.78 | 8.841 | 0.996 |
| S3D0 | 933.025 | 928.776 | 8.282 | 0.993 |
| S3D7 | 944.195 | 955.239 | 8.365 | 0.994 |
| S3D30 | 981.093 | 996.866 | 8.711 | 0.996 |
| S3D60 | 934.637 | 953.617 | 8.061 | 0.992 |
| S4D0 | 935.979 | 939.979 | 8.339 | 0.994 |
| S4D7 | 938.776 | 950.213 | 8.311 | 0.994 |
| S4D30 | 912.088 | 910.51 | 8.407 | 0.994 |
| S4D60 | 737.724 | 741.769 | 7.557 | 0.989 |

**Table S2.** Alpha diversity index of bacteria in waters

| Sample | ACE | Chao1 | Shannon | Simpson |
| --- | --- | --- | --- | --- |
| W0D0 | 360.707 | 368.036 | 5.94 | 0.961 |
| W0D7 | 416.358 | 416.8 | 5.751 | 0.94 |
| W0D30 | 459.495 | 474.303 | 6.506 | 0.971 |
| W0D60 | 377.756 | 379.889 | 6.816 | 0.985 |
| W1D0 | 345.71 | 347.375 | 6.104 | 0.962 |
| W1D7 | 406.497 | 410.312 | 6.268 | 0.97 |
| W1D30 | 453.251 | 451.63 | 5.937 | 0.955 |
| W1D60 | 428.714 | 422.366 | 7.087 | 0.989 |
| W2D0 | 372.329 | 383.115 | 6.087 | 0.963 |
| W2D7 | 315.02 | 310.516 | 5.859 | 0.966 |
| W2D30 | 422.162 | 426.886 | 5.989 | 0.948 |
| W2D60 | 392.908 | 392.121 | 6.855 | 0.986 |
| W3D0 | 297.193 | 298.8 | 5.934 | 0.956 |
| W3D7 | 235.714 | 230.44 | 4.979 | 0.942 |
| W3D30 | 375.279 | 389.037 | 5.294 | 0.92 |
| W3D60 | 396.95 | 394.636 | 6.793 | 0.985 |
| W4D0 | 309.501 | 310.12 | 6.023 | 0.966 |
| W4D7 | 267.506 | 278.286 | 5.34 | 0.954 |
| W4D30 | 328.551 | 351.556 | 5.902 | 0.965 |
| W4D60 | 334.281 | 337.2 | 6.068 | 0.965 |
